# Mood states, gastrointestinal symptoms, and renal biomarkers in Brazilian bodybuilders across different competition preparation phases

**DOI:** 10.7717/peerj.21465

**Published:** 2026-07-01

**Authors:** Douglas Leão Peixoto, Ronaldo Ferreira Moura, Fernando Noronha Almeida, Wilson Max Almeida Monteiro de Moraes, Emanuelle Fernandes Prestes, Bruno Magalhães de Castro, Allan Felipe da Silveira Barros, Dahan da Cunha Nascimento, Gabriel Monaco Maique, Vicente Augusto Castro, Silvio Roberto Barsanulfo Junior, Nathany Ribeiro Barbosa, Pettherson Leonnarddi, Jonato Prestes

**Affiliations:** 1 Physical Education, Catholic University of Brasilia (UCB), Brasilia, Distrito Federal, Brazil; 2 Physical Education, Evangelic University of Goias, Goiânia, Goiás, Brazil; 3 Physical Education, Pontifical Catholic University of Goias, Goiânia, Goiás, Brazil; 4 Physical Education, Claretiano University Center, Goiania, Goiás, Brazil

**Keywords:** Bodybuilding, Longitudinal study, Mood states, Kidney biomarkers, Glomerular filtration rate

## Abstract

This study aimed to examine variations in mood states, gastrointestinal symptoms, and kidney function in non-drug-free amateur bodybuilders across four peri-competition timepoints: 7 days before competition, competition day, 7 days after competition, and 14 days post-competition. The study was characterized as an exploratory longitudinal study with repeated measures. Nine amateur bodybuilders (median age: 30 years) participated in the study. Athletes were assessed at four moments using validated mood and gastrointestinal symptom scales, anthropometric measurements, and biochemical analyses. Blood biomarkers included glucose, urea, creatinine, cystatin C, and glomerular filtration rate. Body mass was recorded at each timepoint to evaluate changes in energy balance. Some mood-related items showed significant changes across the peri-competition phases, including variations in tension, fatigue, anger, and vigor. Body mass increased after the contest, indicating a positive energy balance during recovery. Although group-level biochemical markers remained largely stable, individual analyses revealed relevant fluctuations in glucose, urea, creatinine, and cystatin C. Glomerular filtration rate was lower on the contest day compared to follow-up assessments. The findings highlight the variability in psychological and physiological markers observed in amateur bodybuilders during peri-competition phases, such as mood disturbances, kidney function variations, and post-competition increases in body mass. Coaches and practitioners should closely monitor athletes throughout contest preparation to better support performance, health, and recovery.

## Introduction

Bodybuilding requires significant physical skill, as subjects develop and display their muscularity through precise posing techniques (Peixoto et al., 2024). It involves rigorous training and physical exertion for competition preparation, while competitors are evaluated based on skillful displays of physiques against predetermined standards, similar to other sports (Kind & Helms, 2023). However, non-free drug bodybuilders’ training, pharmacological, dehydration and supplementation practices differ between the off-season and pre-competition periodization phases when compared to other sports or modalities (Almeida et al., 2023; Alves et al., 2020).

In the off-season, the primary goal is muscle hypertrophy through resistance training (Hackett, 2022; Hackett, Johnson & Chow, 2013). As the pre-competition phase begins, about 20 to 30 weeks before a contest, the focus shifts to fat loss while preserving muscle (Hackett, 2022; Hackett, Johnson & Chow, 2013). This often includes increased aerobic exercise or high-intensity interval training for improved fat-loss results (Hackett, 2022; Hackett, Johnson & Chow, 2013). Supplementation in the off-season typically involves protein powder, creatine monohydrate, and an average of five other dietary supplements (Hackett, 2022; Hackett, Johnson & Chow, 2013).

During the off-season, many bodybuilders choose to use anabolic-androgenic steroids (AASs), testosterone in conjunction with compounds such as drostanolone or trenbolone to effectively increase muscle mass (Almeida et al., 2023). As they approach competition, the focus typically shifts towards fat-loss agents, such as clenbuterol, particularly among those who utilize non-natural methods (Hackett, 2022; Hackett, Johnson & Chow, 2013). In the pre-contest phase, bodybuilders concentrate on strategies to reduce body fat while preserving lean muscle through tailored training and nutritional plans (Almeida et al., 2023).

It is important to note, that prolonged use of AASs can lead to certain psychological challenges (Patanè et al., 2020). Users may experience increased irritability or aggressiveness, and mood disorders can arise, resulting in fluctuations that include depressive episodes. Furthermore, difficulties with sleep and heightened anxiety levels are additional aspects that can affect mental well-being (Patanè et al., 2020). Recognizing that these potential effects can promote a greater emphasis on mental health in conjunction with physical goals. Building awareness and implementing safe practices can help athletes navigate these challenges effectively and with less damage.

Aerobic exercise is also increased, though improper management can compromise fat-free mass (Hackett, 2022; Hackett, Johnson & Chow, 2013). Training volume may decrease as athletes prioritize managing fatigue from restricted caloric intake, with intensity often adjusted to accommodate lower energy levels (Alves et al., 2020). Many bodybuilders implement dehydration protocols and reduce carbohydrate intake, further influencing their training approach (Fagerberg, 2018). On the dietary front, athletes follow severe caloric deficits to accelerate fat loss, commonly adopting low-carbohydrate diets to achieve a leaner appearance, while this can affect performance, mood states, gastrointestinal symptoms, and kidney function (de Moraes et al., 2019b; Spendlove et al., 2015).

Also, the relationship between mood and bodybuilder performance is particularly significant (de Moraes et al., 2019b), with research indicating in other sports that mood states can substantially influence performance outcomes (Selmi et al., 2023). Positive mood states, such as high vigor and low levels of tension, depression, anger, and confusion, are associated with better athletic performance, while negative mood states often correlate with poorer outcomes (Selmi et al., 2023). Furthermore, competitive bodybuilding also plays a crucial role in mood regulation, as physiological and psychological functioning is influenced by a variety of stressors, such as competition, hydration status, injuries, diet, training demands, and personal life challenges (Fagerberg, 2018; Masento et al., 2014).

Gastrointestinal symptoms can also significantly impact an individual’s health and athletic performance, with common issues including nausea, vomiting, diarrhea, abdominal pain, flatulence, gastroesophageal reflux, mucosal erosions, and ischemic colitis (de Oliveira, Burini & Jeukendrup, 2014). Research highlights that bodybuilders experience gastrointestinal problems, such as abdominal pain and constipation before and during contest day (de Moraes et al., 2019a). The underlying pathophysiology of these symptoms is multifaceted, involving mechanisms such as ischemia, where strenuous exercise reduces blood flow to the gastrointestinal tract, resulting in mucosal damage and bleeding (de Oliveira, Burini & Jeukendrup, 2014). Intense physical activity prioritizes blood flow to the muscles, reducing gut perfusion and impairing absorption and barrier function (de Oliveira, Burini & Jeukendrup, 2014). Furthermore, dehydration, a practice adopted by bodybuilders, worsens these symptoms by increasing intestinal permeability (de Oliveira, Burini & Jeukendrup, 2014).

Furthermore, bodybuilders adopt various nutritional strategies during different training phases, particularly during bulking and cutting, which can have significant implications on kidney function (Tidmas et al., 2022). Although high-protein diets are not strongly linked to kidney dysfunction in healthy subjects, they may pose risks for those with pre-existing kidney conditions (Tidmas et al., 2022). Hydration practices play a critical role in maintaining health, yet bodybuilders often engage in dehydration during cutting phases, which can potentially harm kidney function (Tidmas et al., 2022). The excessive intake of certain vitamins, such as A, D, and E, poses significant risks, emphasizing the need for careful usage (Tidmas et al., 2022).

The widespread use of AASs among bodybuilders also complicates kidney health, as these substances are well-documented to increase the risk of acute kidney injury (Tidmas et al., 2022). To mitigate these risks, regular monitoring of kidney function is strongly recommended for early detection of potential issues. However, the data collection period usually adopted by previous studies, which compared off-season (12–16 weeks before competition) (Hackett, 2022) with pre-season phases (3–6 weeks before competition) (de Moraes et al., 2019b, 2019a), is different from the present study.

Furthermore, there have been numerous case studies about adverse events associated with bodybuilding practices (Kingston, 2011; Mayr, Domanovits & Laggner, 2012) and less research has focused on the exploration into evaluating variations in reported mood states, gastrointestinal issues, and kidney function. To address the gaps in the current literature, this research marks an innovative exploration into evaluating variations in reported mood states, gastrointestinal issues, and kidney function throughout the 7 days leading up to the contest, on the contest day, 7 days following the contest, and 14 days after the contest. The hypothesis of the present study is that mood states, gastrointestinal symptoms, and kidney function are negatively affected during pre- and post-contest dynamics.

## Methods

### Subjects

Initially, 13 athletes who participated in the Classic Contest Goiania (Brazil) in 2022 were recruited through in-person and coach-in-charge conversations for this study. Athletes completed questionnaires for mood states, gastrointestinal symptoms, and Wisconsin Upper Respiratory Symptom Survey. However, only nine non-drug-free amateur bodybuilders (six men and three women) completed the blood and biochemical analysis and were used for the main results. Therefore, analyses for gastrointestinal symptoms, mood states, and respiratory symptoms were conducted on 13 participants, while biochemical and renal analyses were restricted to nine participants. This study was approved by Institutional Review Board (protocol number: 04463980-5). The research was conducted in accordance with the Declaration of Helsinki (World Medical, 2013), adhered to the guidelines outlined in the Strengthening the Reporting of Observational Studies in Epidemiology (STROBE) statement (von Elm et al., 2008), and all athletes were informed about the study procedures and voluntarily signed an informed consent prior to enrollment.

The inclusion criteria were as follows: (1) competitive male and female bodybuilders aged 18–60 years of any subdivision (*i.e*., men’s physique/classic); (2) ability to provide mood states and gastrointestinal symptoms, and participation in blood analyses during the 7 days pre-contest, contest day, 7 days post-contest, and 14 days post-contest. Given the target population, recruitment was resource-constrained, and we made our best efforts to recruit as many participants as possible within practical limitations (Lakens, 2022).

### Sample size calculation

Given the exploratory nature of this investigation, the sample size was determined by practical constraints in recruiting competitive bodybuilders during the demanding pre- and post-contest period rather than by an *a priori* power calculation. We therefore enrolled all eligible athletes who consented within the study window, yielding nine participants. A sensitivity analysis for paired-samples t-tests indicated that, under this sample size with α = 0.05 and power of 0.80, the minimum detectable effect corresponded to a Cohen’s *d* of 1.06 (large effect) (Lakens, 2022; Helms, Aragon & Fitschen, 2014). The present findings should therefore be regarded as preliminary and hypothesis-generating, intended to guide the design of larger confirmatory studies in this hard-to-recruit population.

### Study procedures

All measurements of gastrointestinal symptom rating Scale, Wisconsin upper respiratory symptom survey, Brunel mood scale, body mass, and biochemical analyses were recorded 7 days before weigh-in, during the contest day, 7 days post-contest and 14 days post-contest.

### Body mass and height

The athletes’ body mass and height were recorded using a digital electronic scale with a 300 kg capacity and an integrated stadiometer (Welmy® W300, Sao Paulo, SP, Brazil). For height measurement, subjects were instructed to take a deep breath and maintain an upright posture.

### Gastrointestinal symptoms

Athletes were invited to complete a modified version of the Abdominal and Epigastric Symptoms Questionnaire (Bovenschen et al., 2006), which included questions about symptoms such as abdominal pain, nausea, vomiting, bloating, regurgitation, loss of appetite, flatulence, abdominal rumbling, belching, heartburn, constipation, and diarrhea. The questionnaire was administered 7 days before weigh-in, during the contest day, 7 days post-contest and 14 days post-contest, with participants rating each symptom on a 1–5 Likert scale, where one indicated “none” and five represented “unbearable.”

### Mood states

Mood states were assessed using the Brunel Mood Scale (BRUMS) questionnaire, which had been translated into Portuguese, and has an initial validation for use in a Brazilian population (Miranda et al., 2008). The BRUMS is a self-administered tool designed to measure six mood dimensions: anger, confusion, depression, fatigue, tension, and vigor. It consists of 24 items, with each dimension represented by four items. Participants rate each item on a five-point scale ranging from 0 (“not at all”) to 4 (“extremely”), selecting the option that best reflects their current feelings or their mood over the past week, including the present day. Example prompts include questions such as “How do you feel now?”, “How have you been feeling in the past week, including today?”, or “How have you been feeling?”

The specific items for each subscale are as follows:

- **Anger**: annoyed, bitter, angry, bad-tempered
- **Confusion**: confused, muddled, mixed-up, uncertain
- **Depression**: depressed, downhearted, unhappy, miserable
- **Fatigue**: worn out, exhausted, sleepy, tired
- **Tension**: panicky, anxious, worried, nervous
- **Vigor**: lively, energetic, active, alert.

### Wisconsin upper respiratory symptom survey

The Portuguese version of the WURSS-21 questionnaire was administered during each phase period (Moreira et al., 2009). Athletes rated the severity of their symptoms using a seven-point Likert scale, where one represented “very lightly,” 3 “lightly,” 5 “moderately,” and 7 “severely.” If a symptom was not present, the corresponding item was marked as 0. A general symptom score was calculated by summing the total severity scores from ten symptom-related questions and nine questions about limitations, collectively categorized as “severity”.

### Blood collection and biochemical analysis

Blood collection and biochemical analysis were conducted at the Standard of Diagnostic Medicine Laboratory in Goiania, Goias (GO), Brazil (Laboratório Padrão Medicina Diagnóstica e Personalizada, 2022). This laboratory participates in three proficiency programs: The Proficiency Testing Program of the Brazilian Society of Clinical Pathology/Laboratory Medicine (PELM), the National Quality Control Program of the Brazilian Society of Clinical Analysis (PNCQ), and the Quality Control and Incentive Program of the Brazilian Society of Pathology (PICQ).

A total of 10 ml of venous blood was meticulously collected from each subject using heparinized tubes. This collection took place following a fasting period of 10–12 h, and was conducted at various intervals: 48 h prior to weigh-in, on the day of the contest, as well as 7 days and 14 days after the contest. To analyze the blood samples, several biochemical parameters were assessed. Phosphate levels (PO_4_) were quantified using the photometric method, while potassium (K) concentrations were determined *via* the selective electrode method. Magnesium (Mg) levels were measured through a colorimetric method. Additionally, glucose was analyzed employing an enzymatic method, urea was assessed using the kinetic ultraviolet method, and creatinine levels were evaluated by another enzymatic approach. Cystatin C was measured utilizing the nephelometry method, and the glomerular filtration rate (GFR) was calculated following the Chronic Kidney Disease Epidemiology Collaboration (CKD-EPI) formula to ensure precise evaluation of renal function (Levey et al., 2003).

Recognizing the significance of health biomarkers in monitoring and diagnosing medical conditions, specific cut-off points have been established. For urea, a level of 43 mg/dL has been set as the threshold (Xue et al., 2014; Fonarow et al., 2005), indicating potential abnormalities in kidney function or hydration status. Glucose levels are evaluated with a cut-off point of 100 mg/dL, which is crucial for identifying risks of diabetes or metabolic disorders (Forti et al., 2019). When it comes to creatinine, different standards apply based on sex: a limit of 1.2 mg/dL is designated for men, while a lower threshold of 1.0 mg/dL is established for women, reflecting physiological differences (Szwarcwald et al., 2019; Zhang et al., 2017). For cystatin, a cut-off value of 0.95 mg/L has been adopted, offering another important indicator for kidney health (Ognibene et al., 2006; Zhang et al., 2017). Finally, for glomerular filtration rate, a cut-off value of 90 mL/min/1.73 m^2^ was used (Levey et al., 2003). Together, these biomarkers play a vital role in comprehensive health assessments.

### Statistical analysis

Because of the low sample size and considering that parametric tests are prone to errors if the data containing outliers or are not normally distributed, we employed a non-parametric repeated-measures Friedman’s Analysis of Variance (ANOVA) between the paired conditions and significant values have been adjusted by the Bonferroni correction for multiple tests. Because of this, the data are presented as mean ranks for tables or as medians and ranges for figures. SPSS, G* Power, and GraphPad were used for statistical analysis. An alpha level of *p* ≤ 0.05 was considered statistically significant for all comparisons.

## Results

During pre- and post-contest competition, no potentially life-threatening adverse events were reported by coaches or athletes. Subjects displayed the following baseline characteristics (expressed as median and range): 30.00 (21.00) years of age; 83.00 (35.60) body mass (kg); and 1.72 (0.26) height (cm). Of the 13 athletes enrolled, all provided complete questionnaire-based and kidney function data (*n* = 13), whereas paired body mass and biochemical assessments (phosphate, potassium, magnesium, and glucose) were available for the subset of nine athletes (*n* = 9). Results are reported separately by sample size, with the relevant *n* stated in the legends of each table and figure.

### Gastrointestinal symptom rating scale

A Friedman test was run to determine if there were differences in gastrointestinal symptoms (Table 1). Significant values have been adjusted by the Bonferroni correction for multiple tests, and statistical significance became null for hunger pangs (*p* > 0.05) and rumbling (*p* > 0.05). There were no differences for the other variables (*p* > 0.05).

**Table 1:** Gastrointestinal symptom rating scale expressed by mean rank.

Symptom	7 days before	Contest day	7 days after	14 days after	*p*
Pain or discomfort in upper abdomen	2.56	2.75	2.44	2.25	0.823
Heartburn	2.50	2.31	2.50	2.69	0.883
Acid reflux	2.25	2.06	3.25	2.44	0.098
Hunger pangs	3.44	2.75	1.94	1.88	0.017^a^
Nausea	2.38	2.38	2.88	2.38	0.522
Rumbling	3.44	2.50	1.81	2.25	0.029^a^
Bloating	2.50	2.86	2.00	2.64	0.457
Belching	2.88	2.75	2.56	1.81	0.163
Passing gas	2.81	2.81	2.00	2.38	0.429
Constipation	2.31	2.62	2.75	2.31	0.550
Diarrhea	2.38	2.38	2.62	2.62	0.801
Loose stool	2.75	2.38	2.44	2.44	0.815
Hard stool	2.69	2.69	2.25	2.38	0.723
Urgent bowel movement	2.19	2.31	3.06	2.44	0.286
Sensation of not completely emptying bowel	1.81	2.81	2.56	2.81	0.278

**Note:**

a significant values have been adjusted by the Bonferroni correction for multiple tests and statistical significance became null. Reported analysis by a total of 13 athletes.

### Wisconsin upper respiratory symptom survey

A Friedman test was run to determine if there were differences in the Wisconsin Upper Respiratory Symptom Survey (Table 2). Significant values have been adjusted by the Bonferroni correction for multiple, tests and statistical significance became null for walk, climb stairs, exercise (*p* > 0.05) and live your personal life (*p* > 0.05). There were no differences for the other variables (*p* > 0.05).

**Table 2:** Wisconsin upper respiratory symptom survey—21 expressed by mean rank.

Symptom	7 days before	Contest day	7 days after	14 days after	*p*
How sick do you feel today?	2.39	2.17	2.61	2.83	0.261
Runny nose	2.22	2.39	2.50	2.89	0.496
Plugged nose	2.22	2.28	2.44	3.06	0.167
Sneezing	2.22	2.22	2.72	2.83	0.129
Sore throat	2.33	2.50	2.78	2.39	0.653
Scratchy throat	2.33	2.50	2.72	2.44	0.846
Cough	2.11	2.17	2.89	2.83	0.088
Hoarseness	2.33	2.11	2.67	2.89	0.163
Head congestion	2.39	2.33	2.39	2.89	0.332
Chest congestion	2.56	2.33	2.33	2.78	0.300
Feeling tired	3.39	2.22	2.06	2.33	0.055
Think clearly	2.75	2.25	2.25	2.75	0.261
Sleep well	3.06	2.22	2.22	2.50	0.312
Breathe easily	3.22	2.06	2.22	2.50	0.096
Walk, climb stairs, exercise	3.33	2.11	1.94	2.61	0.016^a^
Accomplish daily activities	3.06	2.22	2.11	2.61	0.149
Work outside the home	2.75	2.31	2.19	2.75	0.502
Work inside the home	2.89	2.28	2.17	2.67	0.329
Interact with others	3.22	2.22	2.11	2.44	0.071
Live your personal life	3.28	2.22	1.94	2.56	0.024^a^
Compared to yesterday	2.39	2.17	2.61	2.83	0.290

**Note:**

a significant values have been adjusted by the Bonferroni correction for multiple tests and statistical significance became null (*p* > 0.05). Reported analysis for a total of 13 athletes.

### Brunel mood scale

Friedman tests on the BRUMS items revealed time-dependent changes within four of the six mood subscales (Table S1). Overall, athletes shifted from a pre-contest profile characterized by elevated tension, fatigue, and anger toward a post-contest profile of reduced negative mood, with vigor transiently increasing on contest day. Confusion and depression did not change across time points (all *p* > 0.05).

After Bonferroni correction, the Tension subscale showed a sustained reduction in anxious at 7 days (*Mdn* = 1.56) and 14 days post-contest (*Mdn* = 1.61) relative to both 7 days before contest day (*p* ≤ 0.011) and contest day itself (*p* ≤ 0.028). The Vigor subscale displayed an acute increase in active on contest day (*Mdn* = 3.22) relative to 7 days before (*p* = 0.011). The Fatigue subscale showed reductions in worn out, exhausted, and tired on contest day and during the post-contest period relative to 7 days before (all *p* ≤ 0.049). The Anger subscale showed reductions in annoyed on contest day and at 7 days post-contest (*p* ≤ 0.011 *vs*. 7 days before) and in bad tempered at 7 days post-contest (*p* = 0.008 *vs*. 7 days before). Pairwise items that lost statistical significance after Bonferroni correction (worried, nervous, energetic, sleepy, bitter) are reported alongside the full set of adjusted *p*-values in Table S1.

Table S1 Brunel Mood Scale (BRUMS) item-level Friedman test results with Bonferroni-adjusted pairwise comparisons across four time points (7 days before contest, contest day, 7 days after contest, 14 days after contest).

Note: ^a^ = significant values have been adjusted by the Bonferroni correction for multiple tests, and statistical significance became null (*p* > 0.05). * = statistical significance compared to 7 days before (*p* ≤ 0.05). ‡ = statistical significance compared to contest day (*p* ≤ 0.05). Reported analysis for a total of 13 athletes.

### Body mass and biochemical analyses

**Figure 1:**
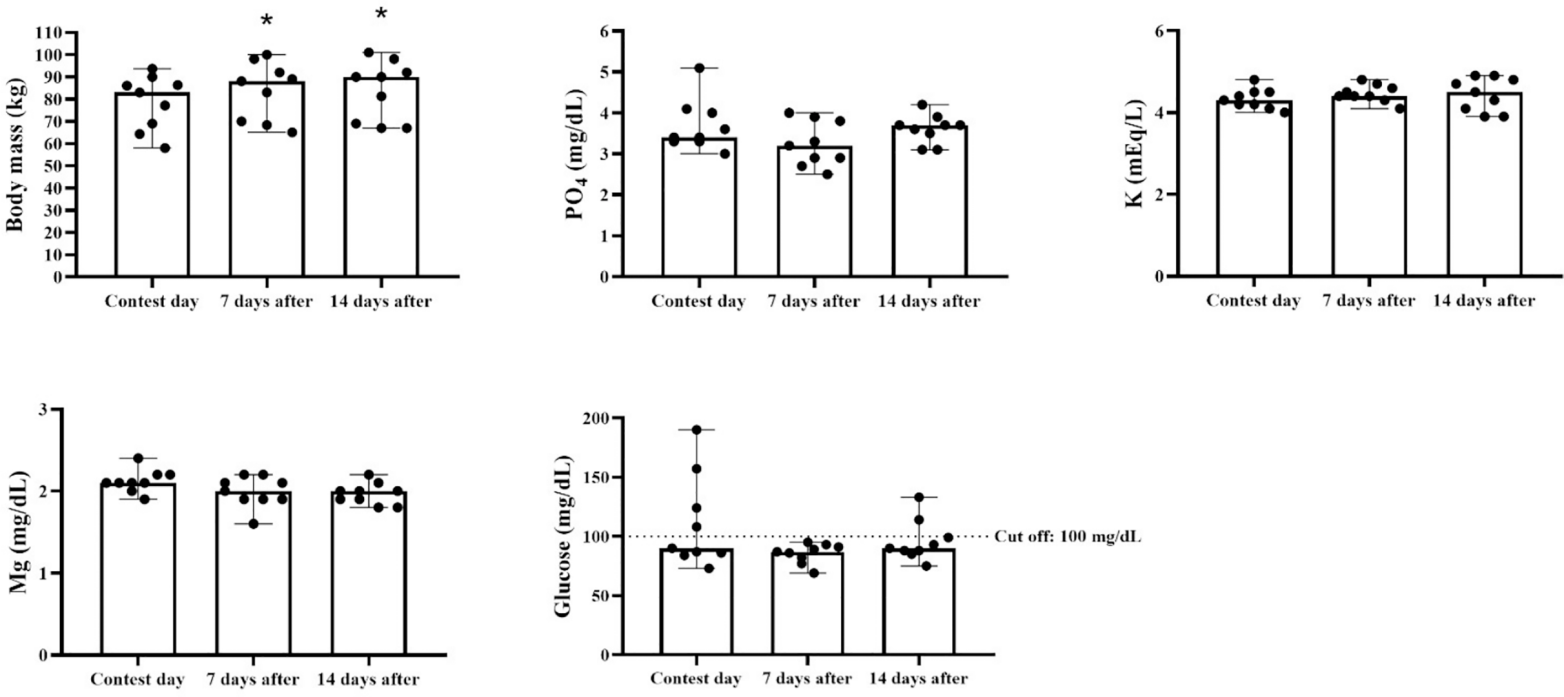
Data presented as medians and ranges for biochemical analysis. Note: * = statistical significance compared to contest day (*p* ≤ 0.05). Reported analysis by a total of nine athletes.

Body mass (kg) statistically increased 7 days after (*Mdn* = 2.33, sig. adjust. = 0.029) and 14 days after (*Mdn* = 2.56, sig. adjust. = 0.007) compared to contest day (Fig. 1). Also, glomerular filtration rate statistically increased 7 days after (*Mdn* = 2.56, sig. adjust. = 0.007) and 14 days after (*Mdn* = 2.33, sig. adjust. = 0.029) compared to contest day. There were no differences for the other variables (*p* > 0.05).

**Figure 2:**
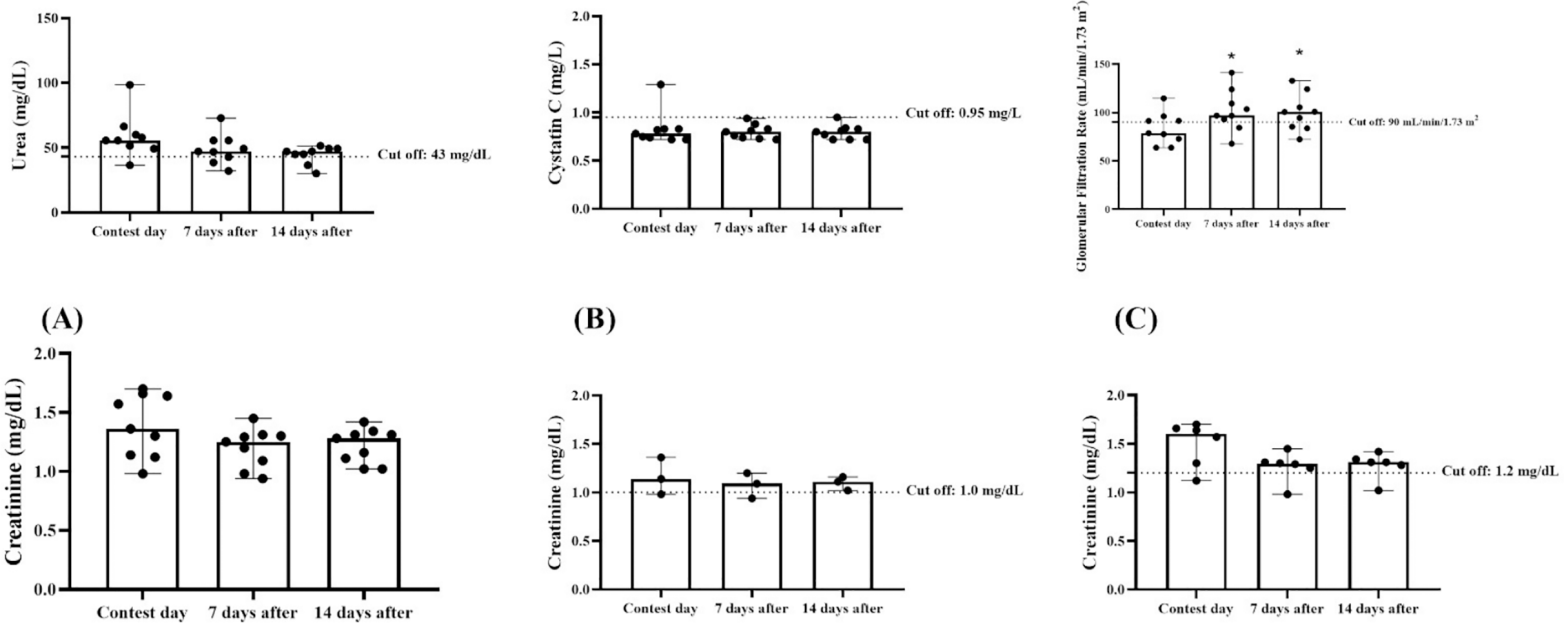
Data presented as medians and ranges for kidney function analysis. Note: * = statistical significance compared to contest day (*p* ≤ 0.05). All participants = (A), female participants (B), male participants (C). Reported analysis by a total of 13 athletes.

In the analysis of metabolic parameters, the following findings were noted among the athletes (Fig. 2): On contest day, four individuals exhibited glucose levels exceeding 100 mg/dL, with two of these maintaining elevated levels 14 days post-contest. Urea measurements revealed that eight participants surpassed the threshold of 43 mg/dL on the contest day, while six did so 7 days later, and seven continued to show elevated levels after 14 days. For cystatin, only one participant recorded a value exceeding 0.95 mg/L during the contest. Regarding creatinine levels, two female athletes presented values above 1.0 mg/dL both on contest day and at the 7-day mark.

Notably, all female participants recorded creatinine levels above 1.0 mg/dL after 14 days. In the male cohort, five participants consistently displayed values greater than 1.2 mg/dL across the contest day, 7 days later, and again 14 days post-contest. Also, five participants had a GFR below 90 mL/min/1.73 m^2^ on contest day, two 7 days later, and three after 14 days.

## Discussion

This longitudinal repeated-measures exploratory study aimed to describe mood states, gastrointestinal symptoms, severity of respiratory infections, and kidney function during four time periods: the 7 days leading up to the contest (a.k.a. peak-week), the contest day itself, the 7 days following the contest, and 14 days post-contest in non-drug-free bodybuilders. Our findings suggest that in this sample of bodybuilders, there were no significant gastrointestinal symptoms or severity of upper respiratory infections observed before or after the contest day. However, mood symptoms showed notable changes.

Anxiety, exhaustion, tiredness, and irritability significantly decreased during the post-competition period, particularly 7 and 14 days after the contest compared with the week preceding the event. These findings suggest a progressive psychological recovery following the competition. In contrast, physical activity scores were higher on the contest day compared with the week prior, which may reflect the acute arousal and activation commonly observed during competitive events. Similar mood fluctuations during competition preparation and recovery have been reported in athletes undergoing intensive training and competition phases (de Moraes et al., 2019b; Lochbaum et al., 2021).

Body mass showed a significant increase 7 and 14 days following the contest day that corroborates with the positive energy balance post competition (de Moraes et al., 2019b), and although there were no statistically significant differences in biochemical health biomarkers, a detailed examination of individual results allowed the identification of individual fluctuations in metabolic biomarkers (Nascimento et al., 2024). Four individuals had glucose levels that exceeded 100 mg/dL, with two of them maintaining elevated levels 14 days after the contest. In terms of urea, eight participants exceeded the threshold of 43 mg/dL on the contest day, while six participants did so 7 days later, and seven continued to show high levels after 14 days. For cystatin, only one individual recorded a value above 0.95 mg/L during the contest. Concerning creatinine levels, two female athletes had values above 1.0 mg/dL on both the contest day and the 7-day follow-up. Significantly, all female participants measured creatinine levels over 1.0 mg/dL after 14 days. In the male group, five participants consistently registered values higher than 1.2 mg/dL on the contest day, 7 days later, and again 14 days post-contest. For the glomerular filtration rate, the values were significantly lower on contest day compared to seven and 14 days after, and some participants displayed values below 90 mL/min/1.73 m^2^.

This research utilized four distinct data collection periods that differ from earlier studies, which compared off-season (12–16 weeks before competition) (de Moraes et al., 2019b) with pre-season phases (3–6 weeks before competition) (Hackett, 2022; de Moraes et al., 2019b, 2019a). No significant differences in gastrointestinal symptoms were noted in this research. In contrast to a prior study (de Moraes et al., 2019a), abdominal pain and constipation were frequently reported by bodybuilders 2 days before weighing. Besides, elevated scores for negative mood fatigue were observed, accompanied by lower scores for positive mood vigor 2 days before weighing and period between weighing and contest day (de Moraes et al., 2019a). In this investigation, heightened levels of anxiety, fatigue, and anger were recorded 7 days prior to and on the day of the contest. Supporting these results, competitive bodybuilding plays a crucial role in mood regulation, as physiological and psychological functioning is influenced by a variety of stressors, such as competition, hydration status, injuries, diet, training demands, and personal life challenges (Fagerberg, 2018; Masento et al., 2014).

In this study, no statistically significant differences were found in the severity of respiratory infections as assessed by the Wisconsin Upper Respiratory Symptom Survey. A previous study (de Moraes et al., 2019b) indicated that during the pre-contest period (3–5 weeks before competition), increased exercise training and restrictive diets were associated with heightened oxidative stress and inflammation. These factors may worsen the severity of upper respiratory tract infections and negatively affect mood states. Although, the Wisconsin Upper Respiratory Symptom Survey serves as an effective tool for evaluating the severity and impact of acute upper respiratory infections, such as the common cold, the addition of blood collection and biochemical analysis improve performance monitoring and provide more reliable control over performance.

In the present study, the glomerular filtration rate was significantly lower on the contest day compared with measurements obtained 7 and 14 days later. However, these findings should be interpreted with caution, as renal biomarkers such as creatinine and glomerular filtration rate are strongly influenced by factors highly relevant in this population, including acute dehydration, dietary manipulation, and particularly high muscle mass (Tidmas et al., 2022).

Thus, rather than indicating renal dysfunction, these variations may reflect acute and reversible physiological responses associated with peak-week strategies commonly used in bodybuilding.

Although some athletes reported the use of performance-enhancing substances and pharmacological agents commonly associated with bodybuilding preparation, such as anabolic-androgenic steroids and diuretics, these variables were not systematically measured or controlled in the present study. Previous literature suggests that substances such as AAS, ACE inhibitors (*e.g*., enalapril), potassium-sparing diuretics (*e.g*., spironolactone and amiloride), as well as dehydration strategies and high intake of certain vitamins, may influence renal biomarkers and fluid balance (Almeida et al., 2023; Tidmas et al., 2022; Ozkurt et al., 2023). However, in the absence of direct measurements, any potential contribution of these practices to the renal biomarker variability observed in this cohort should be considered speculative and interpreted with caution. Therefore, these uncontrolled factors may have substantially influenced the observed physiological responses, limiting the ability to attribute changes to competition phases alone.

Although no statistically significant differences were observed in this investigation for biochemical analysis, the individual responses bring potential clinical significance to the findings (Nascimento et al., 2024). On the contest day, four individuals exhibited glucose levels exceeding 100 mg/dL, with two maintaining elevated levels 14 days post-contest. Bodybuilders’ carbohydrate-loading practices may explain the elevated glucose levels on contest day (de Moraes et al., 2019a). However, previous studies have reported increased oxidative stress and inflammation during contest preparation (de Moraes et al., 2019b). Although these mechanisms were not directly assessed in the present study, they represent plausible physiological pathways that may partially explain the elevated glucose levels observed in some participants, particularly in the context of acute dietary manipulation and competition-related stress (Donath & Shoelson, 2011).

Urea measurements showed that most participants still had elevated levels of renal function markers 14 days after the competition, such as creatinine. Blood urea nitrogen and creatinine represent important predictors of mortality (Fonarow et al., 2005; Aronson & Burger, 2010), however several studies consistently demonstrate that cystatin C performs at least as well as serum creatinine as a renal marker in the adult population, and that it is more sensitive to small changes in glomerular filtration rate than serum creatinine (Ognibene et al., 2006). Even so, all those acute changes in glomerular filtration rate and renal markers raise some concern that bodybuilding practices should be better monitored by athletes and coaches, as well as medical professionals. Thus, regular testing could provide more accurate measures of initial kidney function of athletes and can be used to identify previous unknown underlying conditions or significant changes during differing training phases, which could increase the risk of renal decline (Tidmas et al., 2022). These findings highlight variability in renal biomarkers during peri-competition phases and warrant further investigation.

The present study exhibits several notable limitations, including a limited sample size of male and female bodybuilders and the potential for selection bias. These factors increase the likelihood of including outliers and subsequently introduce bias that may affect the external validity of the findings. Additionally, the outcomes of biochemical assessments and glomerular filtration rate may be influenced by the use of nonsteroidal anti-inflammatory medications, while serum creatinine levels can be affected by factors such as muscle mass and age (Jones et al., 1998).

Another important limitation is the lack of detailed information regarding pharmacological ergogenic aids, hydration strategies, and nutritional practices. Due to time constraints and the competitive context, these variables could not be systematically recorded.

This limitation restricts the interpretation of the observed physiological changes, as these factors may have significantly influenced mood states, gastrointestinal symptoms, and renal biomarkers.

## Conclusion

**Figure 3:**
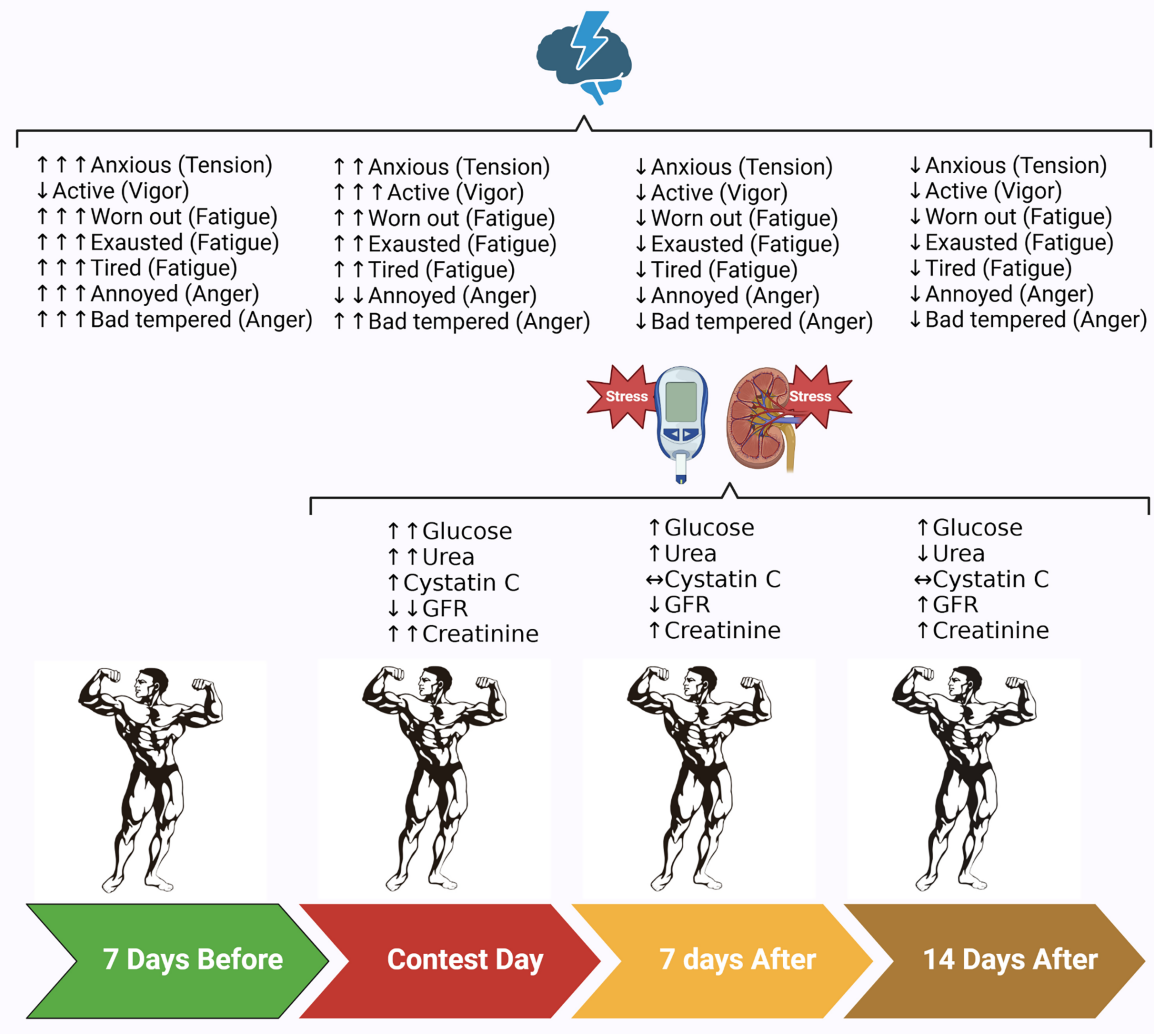
Summary of the main findings of the article.

In summary, this study found no significant gastrointestinal or upper respiratory symptom changes before or after contest day. Mood states demonstrated clear temporal variation across peri-competition phases, with higher tension and fatigue-related scores before and on contest day and lower values during recovery (Fig. 3). Body mass increased significantly 7 and 14 days post-contest, reflecting recovery-phase shifts in energy balance (Fig. 3). Although most biochemical markers did not show statistically significant group-level differences, directional changes across phases were observed for glucose, urea, creatinine, cystatin C, and glomerular filtration rate (Fig. 3). Glomerular filtration rate values were lower on contest day compared to 7 and 14 days after (Fig. 3). These findings highlight measurable physiological variability during peri-competition phases and reinforce the importance of individualized monitoring in competitive bodybuilding contexts.

## Supplemental Information

10.7717/peerj.21465/supp-1 Supplemental Information 1 Gastrointestinal Symptom Rating Scale expressed by mean rank. Note : a = significant values have been adjusted by the Bonferroni correction for multiple tests and statistical significance became null. Reported analysis by a total of 13 athletes.

## Additional Information and Declarations

### Data Availability

The following information was supplied regarding data availability:

The data is available at Figshare:

Castro, Vicente (2025). Raw data from Mood States, Gastrointestinal Symptoms, and Kidney Function in Brazilian Bodybuilders: A Cross-Sectional Analysis of Pre- and Post-Contest Competition. figshare. Dataset. https://doi.org/10.6084/m9.figshare.30913817.v1.
