# Supplementary material for: Mood states, gastrointestinal symptoms, and renal biomarkers in Brazilian bodybuilders across different competition preparation phases
Source: PeerJ. 2026 Jul 1;14:e21465. doi: 10.7717/peerj.21465 (PMC13332715; doi:10.7717/peerj.21465)
Supplement: Supplemental Information 1 — Note : a = significant values have been adjusted by the Bonferroni correction for multiple tests and statistical significance became null. Reported analysis by a total of 13 athletes. [file peerj-14-21465-s001.docx]

Supplementary Table S1**.** Brunel Mood Scale (BRUMS) item-level Friedman test results with Bonferroni-adjusted pairwise comparisons across four time points (7 days before contest, contest day, 7 days after contest, 14 days after contest).

| **Symptom** | **7 days before** | **Contest day** | **7 days after** | **14 days after** | ***P*** |
| --- | --- | --- | --- | --- | --- |
| **Tension** |  |  |  |  |  |
| Panicky | 2.56 | 2.78 | 2.33 | 2.33 | 0.300 |
| Anxious | 3.50 | 3.33 | 1.56*‡ | 1.61*‡ | 0.001 |
| Worried | 3.22 | 2.61 | 2.17 | 2.00 | 0.020^a^ |
| Nervous | 3.28 | 3.06 | 1.72 | 1.94 | 0.004^a^ |
| **Vigor** |  |  |  |  |  |
| Lively | 2.28 | 3.11 | 2.56 | 2.06 | 0.222 |
| Energetic | 1.83 | 3.33 | 2.61 | 2.22 | 0.049^a^ |
| Active | 1.33 | 3.22* | 2.78 | 2.67 | 0.005 |
| Alert | 2.94 | 2.72 | 2.06 | 2.28 | 0.297 |
| **Confusion** |  |  |  |  |  |
| Confused | 2.44 | 2.67 | 2.44 | 2.44 | 0.392 |
| Muddled | 2.78 | 2.50 | 2.44 | 2.28 | 0.392 |
| Mixed-up | 3.00 | 2.67 | 2.28 | 2.06 | 0.101 |
| Uncertain | 2.89 | 2.44 | 2.44 | 2.22 | 0.392 |
| **Fatigue** |  |  |  |  |  |
| Worn out | 3.94 | 2.50 | 1.67* | 1.89* | 0.001 |
| Exhausted | 3.61 | 2.61 | 1.89* | 1.89* | 0.001 |
| Sleepy | 2.94 | 1.94 | 3.00 | 2.11 | 0.034^a^ |
| Tired | 3.67 | 2.06* | 1.94* | 2.33 | 0.006 |
| **Depression** |  |  |  |  |  |
| Depressed | 2.44 | 2.44 | 2.44 | 2.67 | 0.392 |
| Downhearted | 2.78 | 2.28 | 2.28 | 2.67 | 0.491 |
| Unhappy | 2.89 | 2.22 | 2.44 | 2.44 | 0.284 |
| Miserable | 2.78 | 2.33 | 2.33 | 2.56 | 0.300 |
| **Anger** |  |  |  |  |  |
| Annoyed | 3.83 | 1.94* | 1.83* | 2.39 | 0.001 |
| Bitter | 3.50 | 2.22 | 2.06 | 2.22 | 0.001^a^ |
| Angry | 2.94 | 2.50 | 2.28 | 2.28 | 0.066 |
| Bad tempered | 3.72 | 2.33 | 1.78* | 2.17 | 0.002 |

**Note**: ^a^ = significant values have been adjusted by the Bonferroni correction for multiple tests and statistical significance became null (*p* > 0.05). * = statistical significance compared to 7 days before (*p* ≤ 0.05). ‡ = statistical significance compared to contest day (*p* ≤ 0.05). Reported analysis for a total of 13 athletes.
